# Deciphering Insomnia: Benchmarking Automated Sleep Staging Algorithms for Complex Sleep Disorders

**DOI:** 10.1111/jsr.70048

**Published:** 2025-03-27

**Authors:** Umaer Hanif, Anis Aloulou, Flynn Crosbie, Paul Bouchequet, Mounir Chennaoui, Thomas Andrillon, Damien Leger

**Affiliations:** ^1^ VIFASOM, (Vigilance Fatigue Sommeil et Santé Publique) Université Paris Cité Paris France; ^2^ Data Science BioSerenity Paris France; ^3^ APHP, Hôtel‐Dieu Centre du Sommeil et de la Vigilance Paris France; ^4^ IRBA (Institut de Recherche biomédical Des armées) Brétigny France; ^5^ Sorbonne Université Institut du Cerveau ‐ Paris Brain Institute ‐ ICM, Inserm, CNRS, APHP, Hôpital de la Pitié Salpêtrière Paris France

**Keywords:** automated sleep staging, chronic insomnia, machine learning, polysomnography

## Abstract

Polysomnography (PSG) is essential for diagnosing sleep disorders, but its manual interpretation is labor‐intensive. Automated sleep staging algorithms are promising, yet their utility in complex sleep disorders such as insomnia remains uncertain. This study evaluates five of the most recognised sleep staging classifiers—U‐Sleep, STAGES, GSSC, Luna and YASA—on PSG data from 904 patients with chronic insomnia. Performance was assessed using F1 scores, confusion matrices and predicted sleep metrics. The effect of demographics, sleepiness and PSG metrics on each classifier's performance was assessed using linear regression. Across all sleep stages, GSSC performed best (macro F1 score = 0.66), followed by U‐Sleep (0.62), Luna (0.56), STAGES (0.54) and YASA (0.52). GSSC achieved the highest F1 scores in Wake (0.83), N1 (0.22), N2 (0.80), N3 (0.71) and REM (0.76), while U‐Sleep matched its performance in N1 and REM and Luna in N3. STAGES performed poorest in N3 (0.39) and YASA in REM (0.35). Common misclassifications included N1 vs. Wake/N2 and N3 vs. N2, with REM misclassified as Wake/N1/N2 by STAGES, Luna and YASA. GSSC and U‐Sleep exhibited minimal demographic bias, while STAGES and Luna had more. No performance difference was observed between chronic insomnia patients with and without abnormal PSG. Sleep metric accuracy was highest for U‐Sleep (TST, *R* ^2^ = 0.88), STAGES (SOL, *R* ^2^ = 0.82) and GSSC (WASO, *R* ^2^ = 0.82). These findings underscore the solid yet variable performance of the classifiers and highlight GSSC and U‐Sleep as leading tools for sleep staging in patients with chronic insomnia.

## 1 Introduction

After the first discoveries of rapid eye movement (REM) sleep in the 1950s (Aserinsky and Kleitman 1953; Dement and Kleitman 1957; Jouvet 1965), a consensus on how to analyse sleep stages has been the subject of numerous discussions, rules and publications to harmonise its interpretation for clinicians and researchers worldwide. From the seminal manual by Rechtschaffen and Kales (Rechtschaffen and Kales 1968), to the regularly updated standards of the American Academy of Sleep Medicine (AASM) (Iber et al. 2007; Berry et al. 2012; Berry et al. 2017), the challenge has been to analyse sleep uniformly across different sleep centres to enable meaningful comparisons of clinical and research findings.

Sleep staging involves segmenting sleep into distinct stages—wakefulness, REM sleep and non‐REM sleep (the latter comprising stages N1, N2 and N3)—based on physiological markers (Berry et al. 2012). These stages provide insights into sleep architecture and are crucial for diagnosing and managing sleep disorders (*International Classification of Sleep Disorders* 2014). Accurate sleep staging helps clinicians assess sleep quantity and quality by using common sleep metrics (such as total sleep time [TST], sleep onset latency [SOL] and wake after sleep onset [WASO]), understand sleep‐related abnormalities and develop personalised interventions for patients (Boulos et al. 2019). Sleep staging can also provide insights into changes in sleep architecture associated with treatments, such as pharmacological interventions (Food and Drug Administration: US Department of Health E and W 1997; European Medicines Agency 2011). Finally, it plays a central role in basic research by allowing an objective and data‐driven definition of sleep across human and animal models.

The field of sleep medicine has evolved considerably with the digitisation of recordings, which 50 years ago were traced in pen and ink on kilos of paper (Palagini and Rosenlicht 2011), but the difficulty remains the same: reading thousands of 30‐s epochs, page by page on a screen, for a single patient's night. Traditionally, scoring sleep stages relies on manual interpretation of polysomnographic (PSG) data. While effective, this manual process is time‐consuming, resource‐intensive and prone to variability between scorers (Rosenberg and van Hout 2013; Lee et al. 2022). It is also biased toward features that can be identified by the human eye (Decat et al. 2022). These limitations have motivated the development of automated sleep staging tools, which can reduce cost, enhance consistency and offer a scalable solution to meet the growing demands of sleep medicine and research (Gaiduk et al. 2023).

More recently, advances in machine learning have rapidly benefited the development of algorithms that predict sleep stages. In just a few years, several algorithms have been proposed and compared to human scoring. Algorithms such as Luna (https://zzz.bwh.harvard.edu/luna/ n.d.) and YASA (Vallat and Walker 2021) rely on classical feature extraction and tree‐based machine learning methods, while algorithms such as SleepNet (Biswal et al. 2017), DeepSleepNet (Supratak et al. 2017), STAGES (Stephansen et al. 2018), U‐Sleep (Perslev et al. 2021) and GSSC (Hanna and Flöel 2023) utilise convolutional neural networks (CNNs) (Gu et al. 2018), sometimes combined with recurrent neural networks (RNNs) (Yu et al. 2019) (Table 1), to classify sleep stages based on signals from electroencephalography (EEG), electrooculography (EOG) and electromyography (EMG), each of which captures unique aspects of sleep physiology. These methods, as well as attention‐based methods, such as SeqSleepNet (Phan et al. 2019) and SleepTransformer (Phan et al. 2022) are validated primarily on publicly available datasets, which contain data from non‐clinical populations. Some of these algorithms have reported high agreement with manual scoring in controlled datasets. However, validation on other clinical populations, such as insomnia, remains limited, underscoring the need for studies that test these algorithms in broader, more diverse settings.

**TABLE 1:** Summary of the sleep staging classifiers investigated in this study.

Model	Architecture	Input channels	Datasets	Performance
STAGES (Stephansen et al. 2018)	CNN (Gu et al. 2018) and RNN (Yu et al. 2019)	EEG: C3/C4 & O1/O2 EOG: LEOG & REOG EMG: Chin	4 cohorts 2784 PSGs (train) 793 PSGs (test)	0.78 ± 0.06 (DH; Guillot et al. 2020) 0.70 ± 0.16 (DO; Guillot et al. 2020) 83.4 ± 7.1% (TS)
Luna (https://zzz.bwh.harvard.edu/luna/ n.d.)	Feature extraction Gradient‐boosted classifier (Natekin and Knoll 2013)	EEG: C3/C4	7 cohorts 3163 PSGs (train) 585 PSGs (test)	Unknown (No paper published)
U‐Sleep (Perslev et al. 2021)	UNet (Ronneberger et al. 2015)	EEG: F1/F2/C3/C4/O1/O2 EOG: LEOG/REOG	16 cohorts 15,660 PSGs (85% train, 15% test)	0.83 ± 0.05 (DH) 0.79 ± 0.10 (DO) 0.79 ± 0.03 (TS)
YASA (Vallat and Walker 2021)	Feature extraction Gradient‐boosted classifier (Natekin and Knoll 2013)	EEG: C3/C4 *EOG: LEOG/REOG* *EMG: Chin*	7 cohorts 3163 PSGs (train) 585 PSGs (test)	0.79 ± 0.11 (DH) 0.74 ± 0.12 (DO) 87.5% (median, TS)
GSSC (Hanna and Flöel 2023)	CNN (ResNet (He et al. 2016)) RNN (GRU (Chung et al. 2014)) Linear layers	EEG: F1/F2/C3/C4 *EOG: LEOG/REOG*	4 cohorts 2983 PSGs (train) 331 PSGs (test)	0.82 ± 0.08 (DH) 0.80 ± 0.11 (DO) Unknown (TS)

*Note*: A slash (/) between two channels indicates that only one is required, whereas an ampersand (&) indicates that both are required. Modalities in italic font are optional. Performance was evaluated using the macro F1 score on the DREEM dataset for healthy participants (DH, *n* = 25) and patients with sleep‐disordered breathing (DO, *n* = 55), based on the consensus of five expert sleep scorers. The performance reported in the original paper for each model on its respective test set (TS) is also provided (accuracy is reported in %, and macro F1 in values between 0 and 1).

*Abbreviations*: CNN—Convolutional neural network, EEG—Electroencephalography, EMG—Electromyography, EOG—Electrooculography, GRU—Gated recurrent unit, PSG—Polysomnography, RNN—Recurrent neural network.

Prior comparative studies have highlighted the varying performance of these algorithms, emphasising the importance of benchmarking them across different populations and conditions. For example, a recent comparative study by Guillot et al. compared several deep learning‐based classifiers, finding performance discrepancies depending on dataset specifics and patient characteristics (Guillot and Thorey 2021). Another study by Chambon et al. evaluated a set of sleep staging algorithms on clinical data, observing that algorithm accuracy often depends on the variability and quality of the physiological signals (Chambon et al. 2018). Finally, Olesen et al. showed that overall classification accuracy increased with more training data and data sources, while demonstrating that different cohorts show varying levels of generalisation to other cohorts (Olesen et al. 2021). These studies underscore that the promise of automated sleep staging depends not only on the architectural sophistication of algorithms but also on validating their generalisability and reliability in real‐world clinical conditions.

This study aims to compare five of the most well‐recognised and validated algorithms for the detection of sleep stages (U‐Sleep (Perslev et al. 2021), STAGES (Stephansen et al. 2018), GSSC (Hanna and Flöel 2023), Luna (https://zzz.bwh.harvard.edu/luna/ n.d.) and YASA (Vallat and Walker 2021)) and estimation of sleep metrics in a well‐referenced and clinically validated dataset of PSGs performed in subjects suffering from chronic insomnia with normal and abnormal PSG results. Patients with chronic insomnia and abnormal PSG recordings frequently exhibit atypical sleep patterns that may affect staging accuracy, such as prolonged wakefulness, more frequent movement artefacts and reduced deep and REM sleep (Baglioni et al. 2014). As for patients with a diagnosis of chronic insomnia but no abnormality in their PSG, there could be more subtle signs of sleep disruptions that algorithms have not been trained on and that could undermine their performance (Stephan and Siclari 2023; Andrillon et al. 2020). Validating automated algorithms on such data is essential for their broader application, as these tools are increasingly seen as foundational to advancing personalised sleep care. By testing and comparing these algorithms on an insomnia‐centric dataset, this study seeks to bridge the gap between innovation and clinical utility, moving automated sleep staging closer to real‐world applications that can benefit patients with sleep disorders now and in the future.

## 2 Methods

### 2.1 Participants

This study extends the cohort from a previously published investigation (Andrillon et al. 2020), which now includes 904 patients with chronic insomnia. Each participant underwent a single night of PSG to evaluate their sleep patterns, which were part of their clinical assessment. All participants provided written consent and approved the use of their data for research purposes. Chronic insomnia was diagnosed in accordance with the International Classification of Sleep Disorders, Third Edition (ICSD‐3) (*International Classification of Sleep Disorders* 2014), defined by persistent difficulties with sleep initiation, maintenance, or early morning awakenings occurring at least three nights per week for a minimum of 3 months (Riemann et al. 2023).

#### 2.1.1 Recruitment Procedure

Subjects who met the criteria for chronic insomnia were retrospectively identified from the Sleep and Vigilance Center at Hôtel‐Dieu Hospital in Paris, France. As part of routine clinical evaluation, they underwent either laboratory‐based or ambulatory PSG to assess their sleep patterns. Independent medical practitioners at the center conducted the diagnostic assessments, separate from the current study. Chronic insomnia subjects were further divided into two groups: those with abnormal PSG (i.e., chronic insomnia with objective findings described below) and those with normal PSG (i.e., chronic insomnia without objective findings).

#### 2.1.2 Chronic Insomnia With Abnormal PSG

There were 690 chronic insomnia patients with abnormal PSG in our dataset. Abnormal PSG was defined following ICSD‐3 criteria, identifying three subtypes: sleep onset insomnia (SOL > 30 min), sleep maintenance insomnia (WASO > 30 min) and early morning awakening insomnia (natural awakening > 1 h before desired wake time) (*International Classification of Sleep Disorders* 2014; Riemann et al. 2023). Participants meeting at least one of these criteria were categorised as having abnormal PSG.

#### 2.1.3 Chronic Insomnia With Normal PSG

There were 214 chronic insomnia patients with normal PSG in our dataset. These individuals reported chronic insomnia but exhibited no objective PSG abnormalities: SOL < 30 min, WASO < 30 min, natural awakening < 1 h before the desired wake time. Participants not meeting any ICSD‐3 criteria, as defined above, were classified as having normal PSG.

#### 2.1.4 Ethics

The research adhered to the ethical guidelines outlined in the Declaration of Helsinki of 1975, revised in 2001. Approval for the retrospective analysis of PSG data from patients was obtained from the local ethics committee (Comité de Protection des Personnes [CPP] Ouest IV, Nantes, France). Patient data were pseudonymised and analysed in compliance with legal regulations set forth by the Commission Informatique et Liberté (CNIL) in France.

### 2.2 PSG Recordings and Sleep Scoring

The study utilised two PSG devices; most studies were recorded with NOX‐A1 (Nox Medical, *N* = 890) and only a few with MORPHEUS (Micromed S.p.A., *N* = 14). The number of EEG, EOG and EMG derivations varied across devices, with respiratory parameters recorded exclusively using NOX devices. Both devices were validated and regularly employed in the sleep centre. After the night recording, PSG data were visually examined and scored according to AASM guidelines (Berry et al. 2012) to produce individual hypnograms. Arousals, leg movements and respiratory events were also identified. Arousals were characterised by EEG acceleration within epochs lasting between 3 and 15 s. A sleep medicine specialist conducted this visual scoring process. Specifically, 30‐s epochs containing EEG (mastoid‐referenced), EMG and EOG data were categorised into wakefulness, NREM stages 1 to 3 (N1, N2, N3) and REM sleep. For this retrospective analysis, PSG files that had been initially scored by someone other than the lab's most experienced sleep technician were re‐scored from scratch by the same technician to ensure consistency across the dataset. Key parameters reflecting sleep macro‐structure were extracted from individual hypnograms, including time in bed (TIB), TST, WASO, SOL, REM onset latency (ROL) and the distribution of sleep stages across the whole night (% N1, % N2, % N3, % REM).

### 2.3 Library Comparison

The aim of this study was to compare different automated sleep scoring algorithms on our cohort of patients with chronic insomnia, to compare their respective accuracy against manually scored hypnograms. We selected five well‐known state‐of‐the‐art algorithms, presented in the order they were released: STAGES (Stephansen et al. 2018), Luna (https://zzz.bwh.harvard.edu/luna/ n.d.), U‐Sleep (Perslev et al. 2021), YASA (Vallat and Walker 2021) and GSSC (Hanna and Flöel 2023); all validated in the scientific community on different cohorts with various sleep disorders. Table 1 summarises the key features of each algorithm: the model architecture, the required (and optional) input channels, the datasets it was trained and tested on and its performance on the publicly available DREEM dataset (Guillot et al. 2020) (split into healthy participants, *n* = 25, and patients with sleep‐disordered breathing, *n* = 55), as well as the performance reported on the test set of each model's original study. The performance values on the DREEM dataset were taken from the GSSC paper (Hanna and Flöel 2023), which, in turn, had sourced the performance values for all classifiers (except GSSC) from the YASA paper (Vallat and Walker 2021).

#### 2.3.1 Stages

The STAGES algorithm was published by researchers at Stanford University in 2018 and was one of the first deep learning‐based sleep stage classifiers, trained and validated on several thousand PSGs from four different cohorts (Stephansen et al. 2018). STAGES takes EEG, EOG and chin EMG channels as inputs and utilises a neural network architecture consisting of CNN (Gu et al. 2018) and long short‐term memory (LSTM) (Yu et al. 2019) layers. This algorithm is capable of scoring sleep reliably at a resolution of 5 s instead of 30‐s epochs and was the first to introduce hypnodensities—a probability distribution of sleep stages. Besides generating sleep stage predictions at a higher level than any individual scorer they compared performance to, the authors also demonstrated that hypnodensities produced by their algorithm enabled the efficient diagnosis of type 1 narcolepsy (Stephansen et al. 2018).

#### 2.3.2 Luna

The Luna algorithm was initially released in 2019 and has since been followed by several updates. Luna is an open‐source C/C++ software package that provides several tools for the analysis of PSG data, including automatic sleep staging, spindle detection and artefact detection (https://zzz.bwh.harvard.edu/luna/ n.d.). Luna uses various handcrafted features derived from EEG (either one of the central channels) which are fed into a gradient boosting model, an ensemble technique that builds a series of decision trees to make predictions (Natekin and Knoll 2013). The model outputs sleep stages in 30‐s epochs. Luna has been trained and validated on 3748 PSG recordings from seven different cohorts from the National Sleep Research Resource (NSRR). Luna has been validated on datasets comprising patients with insomnia, sleep apnea, restless legs syndrome, narcolepsy and paediatric cohorts (Djonlagic et al. 2021; Adra et al. 2022). An initial publication by the developers of Luna is currently in progress.

#### 2.3.3 U‐Sleep

The U‐Sleep algorithm was published in 2021 and represents a robust, generalisable sleep staging classifier, trained and evaluated on a diverse dataset of 15,660 PSGs from 16 cohorts (Perslev et al. 2021). U‐Sleep takes a combination of EEG and EOG channels as input and consists of an encoder, decoder and a segment classifier, based on a series of convolutional neural network layers inspired by the U‐Net architecture (Ronneberger et al. 2015). U‐Sleep allows the user to select the temporal resolution of predictions, making it possible to predict sleep stages every second of a recording if needed. Previous studies have demonstrated U‐Sleep's robustness and high agreement with expert scorers, making it a suitable choice for clinical and research applications. U‐Sleep has been validated on diverse datasets including healthy adults, patients with obstructive sleep apnea, insomnia, narcolepsy and multi‐centre sleep studies (Perslev et al. 2021; Fiorillo et al. 2023; Kevat et al. 2024; Yang et al. 2020; Bechny et al. 2024).

#### 2.3.4 YASA

YASA (Yet Another Sleep Algorithm) was published in 2021 and is a publicly available open‐source software developed for the automatic scoring of sleep stages using EEG recordings. YASA employs a combination of feature extraction and classification techniques to predict sleep stages in 30‐s epochs. It incorporates spectral analysis of EEG signals to identify characteristic features such as slow waves, sleep spindles and REM‐related activities (Vallat and Walker 2021). YASA was trained and evaluated on the same PSG recordings as Luna from the NSRR. YASA has been validated on datasets including healthy individuals, individuals with insomnia, sleep apnea, periodic limb movement disorder and multi‐site cohorts (Vallat and Walker 2021; Benedetti et al. 2023).

#### 2.3.5 GSSC

GSSC (Greifswald Sleep Stage Classifier) is the most recent of our studied classifiers, published in 2023. It takes EEG and EOG channels as inputs and is based on CNNs of the ResNet architecture (He et al. 2016). GSSC was trained and validated on 3314 PSG recordings from four different cohorts (Hanna and Flöel 2023). A benefit of GSSC is that it allows for missing channels, i.e., it can generate predictions using a combination of EEG and EOG channels or using only one or the other. GSSC outputs sleep stage predictions in 30‐s epochs. In their article, the authors compare GSSC to STAGES, U‐Sleep and YASA, claiming superior performance over STAGES and YASA but not statistically significant over U‐Sleep (Hanna and Flöel 2023).

#### 2.3.6 Performance Evaluation

The performance of each algorithm was evaluated using the F1 score for each sleep stage and the macro F1 score (average of sleep stage F1 scores) for overall performance on an epoch‐by‐epoch basis. Statistical differences in performance among the classifiers were assessed using repeated measures ANOVA, followed by post hoc paired *t*‐tests for pairwise comparisons, with Bonferroni correction applied to adjust the p‐values. Furthermore, confusion matrices were utilised to assess the sensitivity of each classifier in each sleep stage and to explore which sleep stages were confused the most with one another. Sleep metrics (TST, SOL and WASO) were estimated based on each classifier's predictions and compared to gold‐standard sleep metrics derived from technicians' annotations. Finally, bias in each algorithm was examined by using univariate linear regression models to predict the macro F1 score of each classifier based on demographic and PSG variables.

## 3 Results

**FIGURE 1:**
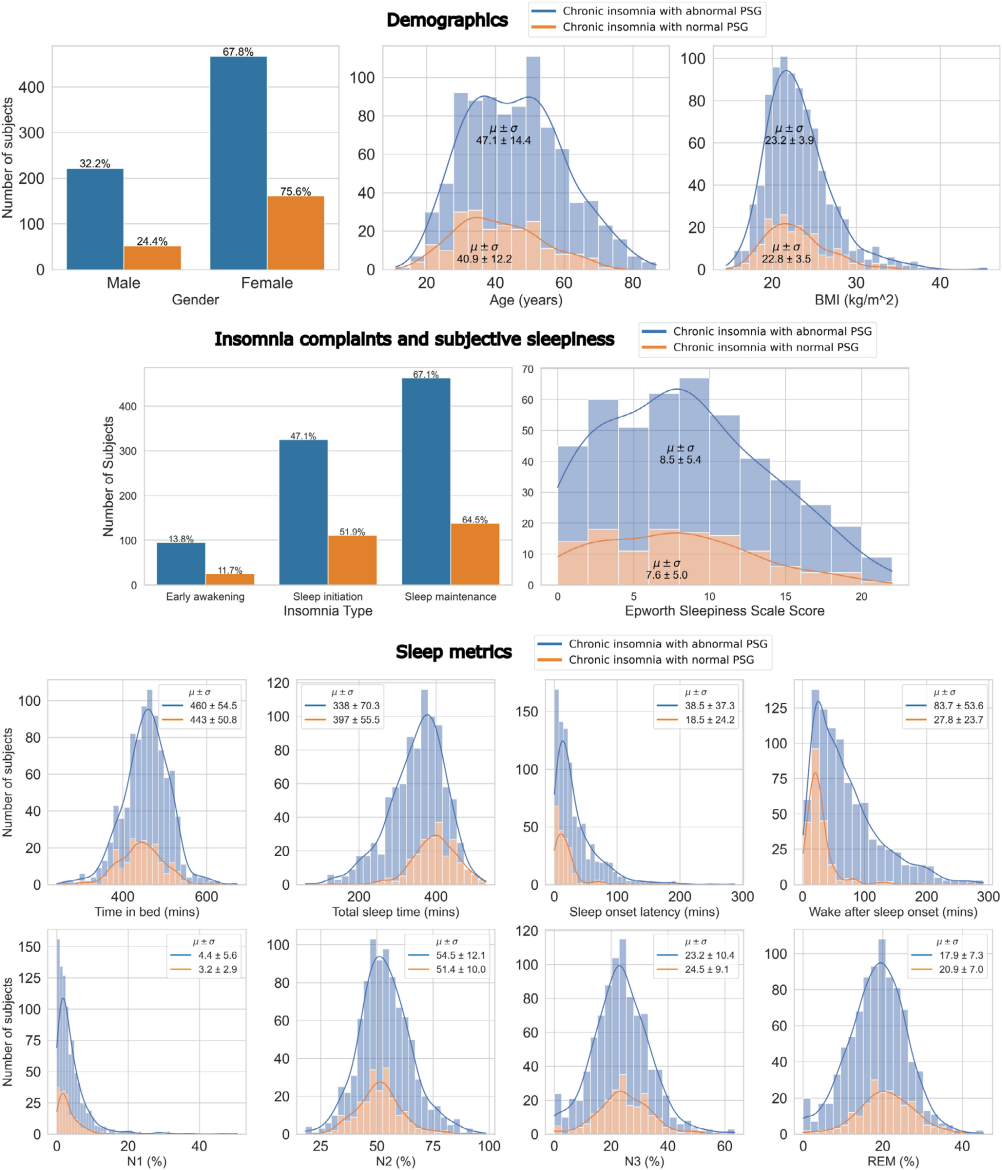
Characteristics of patients with chronic insomnia with abnormal polysomnography (PSG, blue) and normal PSG (orange). The variables shown for each group include demographics (gender, age, BMI), insomnia subtypes (sleep initiation, sleep maintenance, early awakening), subjective sleepiness (Epworth Sleepiness Scale) and sleep metrics from PSG (time in bed, total sleep time, sleep onset latency, wake after sleep onset and % sleep stages).

The study population consisted of 904 patients, with 628 being female (69.5%) and having a mean age of 45.7 ± 14.1 years (range 11–87 years) and a mean BMI of 23.1 ± 3.8 kg/m^2^. Of the 904 patients, 690 had abnormal PSG results (SOL > 30 min and/or WASO > 30 min and/or natural awakening > 1 h before desired wake time) and 214 had normal PSG results (SOL < 30 min, WASO < 30 min and natural awakening < 1 h before desired wake time). Figure 1 shows the distribution of demographic variables, self‐reported insomnia complaints and sleepiness and objective sleep metrics from PSG for each of the two groups. Table S1 in the Supplement contains further information about comorbidities, medications and smoking.

**FIGURE 2:**
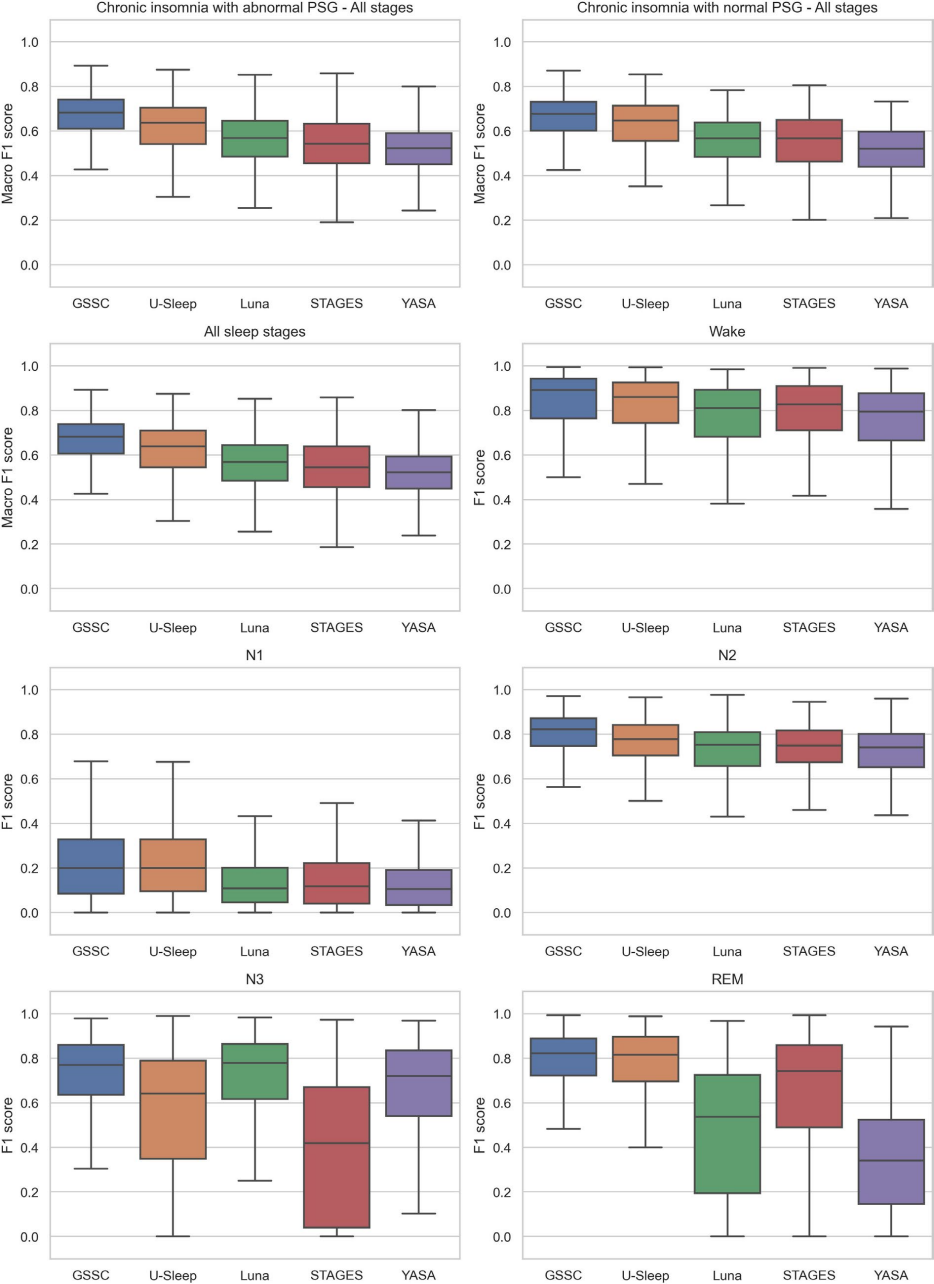
F1 score for five different sleep staging classifiers (GSSC, U‐Sleep, Luna, STAGES, YASA) with respect to all sleep stages (average or macro F1), and individual sleep stages (Wake, N1, N2, N3 and REM sleep).

Figure 2 shows boxplots of the overall performance divided into with and without abnormal PSG groups, then across all subjects, and finally, the performance in each individual sleep stage (Wake, N1, N2, N3, REM). The mean macro F1 scores across all stages (and mean F1 scores for individual stages) were: 0.66 for GSSC (Wake: 0.83, N1: 0.22, N2: 0.80, N3: 0.71, REM: 0.76); 0.62 for U‐Sleep (Wake: 0.81, N1: 0.22, N2: 0.76, N3: 0.56, REM: 0.76); 0.56 for Luna (Wake: 0.76, N1: 0.13, N2: 0.72, N3: 0.70, REM: 0.47); 0.54 for STAGES (Wake: 0.78, N1: 0.14, N2: 0.73, N3: 0.39, REM: 0.64) and 0.52 for YASA (Wake: 0.75, N1: 0.12, N2: 0.72, N3: 0.64, REM: 0.35). Furthermore, the weighted F1 scores (considering the proportion of each sleep stage) were: 0.91 for GSSC, 0.90 for U‐Sleep, 0.88 for Luna, 0.87 for STAGES and 0.87 for YASA. Table S2 in the Supplement provides Bonferroni‐corrected p‐values obtained from pairwise comparisons of all classifiers' performances using pairwise t‐tests, both overall and within each sleep stage. All classifiers differed significantly from each other in overall performance and Wake. In N1, only GSSC vs. U‐Sleep and Luna vs. STAGES were not significantly different; in N2, only Luna vs. STAGES and Luna vs. YASA; in N3, only GSSC vs. Luna; and in REM, only GSSC vs. U‐Sleep.

Thus, GSSC performed the best overall and in each individual sleep stage, although U‐Sleep achieved the same performance in stages N1 and REM. Furthermore, U‐Sleep had the second highest performance in Wake and N2 but only performed better than STAGES in N3. Luna achieved the highest performance in N3 along with GSSC but showed moderate performance in all other stages compared to the other classifiers. STAGES had the worst performance in N3 of any classifier, while placing itself in the middle for stages Wake, N1, N2 and REM. Finally, YASA showed the poorest performance in Wake, N1, N2 and REM, although Luna had the same performance in N2.

**FIGURE 3:**
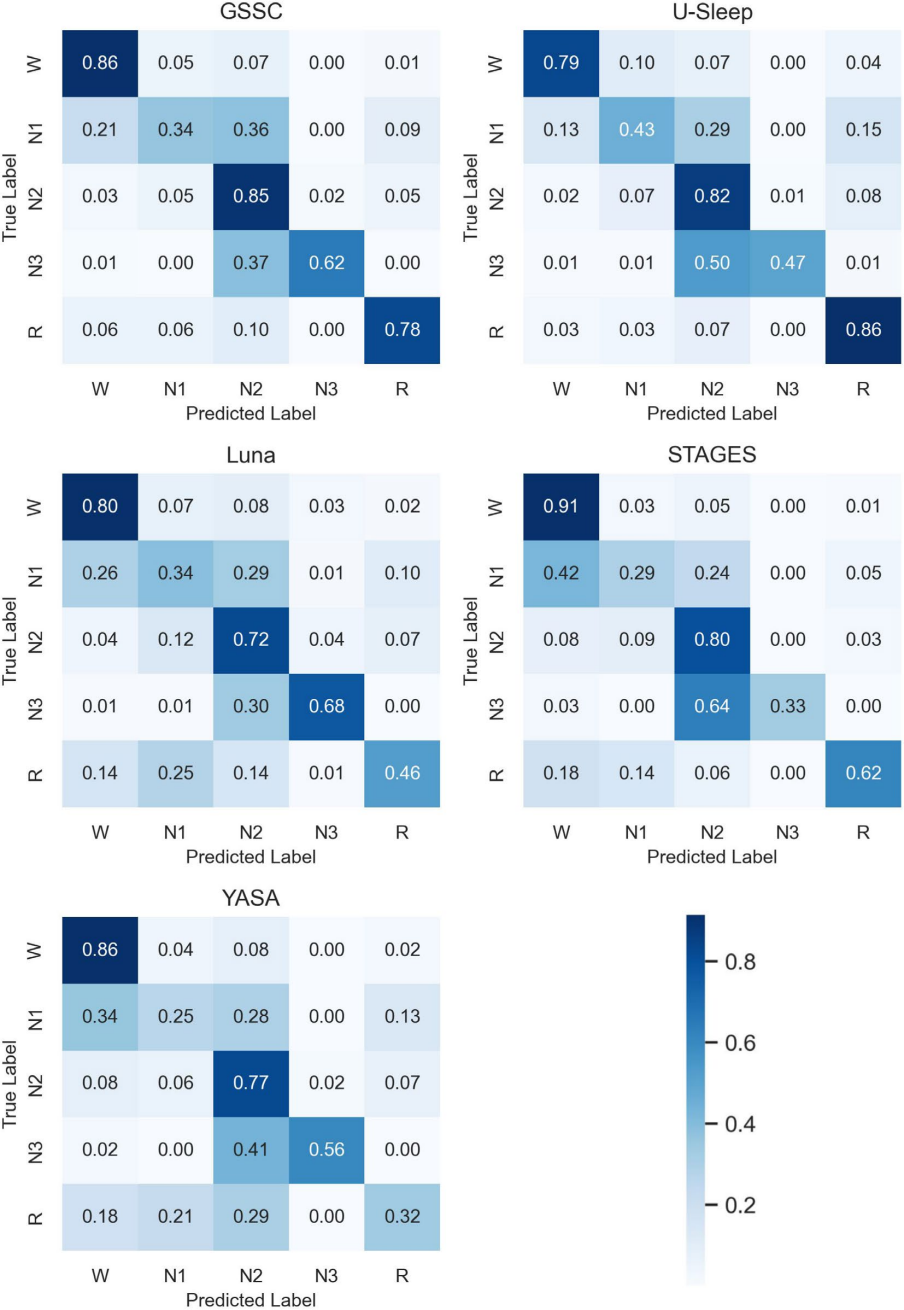
Confusion matrices for five different sleep staging classifiers (GSSC, U‐Sleep, Luna, STAGES, YASA) with respect to true and predicted sleep stages (W, N1, N2, N3, R). W—Wake, R—REM.

Figure 3 shows the confusion matrices for the different classifiers by performing an epoch‐by‐epoch comparison with the gold‐standard annotations. STAGES had the highest sensitivity in detecting Wake (91%), U‐Sleep in N1 (43%), GSSC in N2 (85%), Luna in N3 (68%) and U‐Sleep in REM (86%). Furthermore, GSSC showed the second highest sensitivity (shared with YASA in Wake and Luna in N1) in all stages except for N2, where it had the highest sensitivity of all classifiers. Across all classifiers, Wake was mainly confused with N1 and N2 and rarely with N3 and REM. Similarly, N1 was mainly confused with Wake and N2 by all classifiers, although U‐Sleep also confused N1 with REM more often than Wake. Only STAGES and YASA confused N1 more frequently with Wake than N2. For N2, all classifiers mainly confused this stage with Wake, N1 and REM, and less so with N3. For N3, all classifiers confused this stage almost exclusively with N2, with STAGES misclassifying the majority of N3 epochs as N2 (64%), contributing to its poor performance in N3. U‐Sleep also struggled with correct detection of N3 by misclassifying 50% of N3 epochs as N2. Finally, REM was confused most with N2 by GSSC, U‐Sleep and YASA, whereas REM was confused most with N1 by Luna and Wake by STAGES. Consistent across all classifiers is the fact that REM was never confused with N3 (except a few epochs by Luna).

**FIGURE 4:**
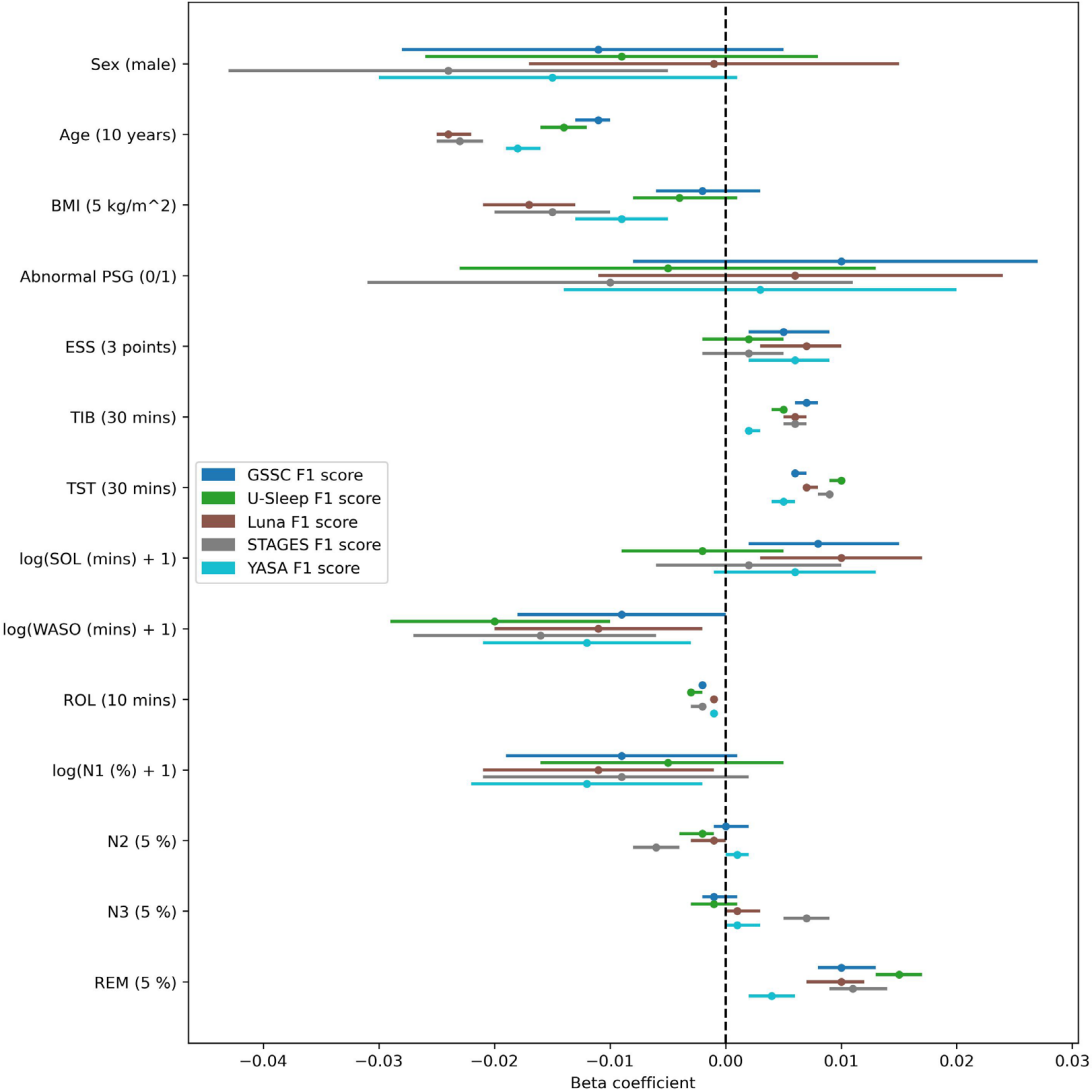
Beta coefficients obtained from univariate linear regression models used to predict each sleep stage classifier's macro F1 score using demographic and polysomnography (PSG) variables. The unit increase for each variable is reported in a parentheses next to the variable. ESS—Epworth Sleepiness Scale, TIB—Time in bed, TST—Total sleep time, SOL—Sleep onset latency, WASO—Wake after sleep onset, ROL—REM onset latency.

Figure 4 illustrates the impact of demographic variables, subjective sleepiness and PSG metrics on the performance of each sleep staging classifier. Furthermore, Table S3 in the Supplement provides percentages/means of each variable and p‐values for the beta coefficients. Beta coefficients were derived from univariate linear regression models predicting the macro F1 score of each classifier based on individual variables. Among the classifiers, only STAGES exhibited a slight bias toward females in terms of sex. Regarding age, Luna and STAGES showed the strongest bias toward younger individuals, while GSSC and U‐Sleep displayed the least bias, with YASA falling in between. A similar pattern was observed for BMI, though with no bias for GSSC and U‐Sleep. No classifiers demonstrated a relationship with normal versus abnormal PSG results in patients with chronic insomnia. Subjective sleepiness, measured by the Epworth Sleepiness Scale, was positively associated with the performance of Luna, YASA and GSSC, but not with STAGES or U‐Sleep. Among the sleep metrics, all classifiers' performances showed a positive association with TIB, TST and % REM, and a slight negative association with ROL, with YASA showing the weakest association in all cases. For SOL, only GSSC and Luna showed slight positive associations. In the case of WASO, all classifiers exhibited negative associations, although not significant for GSSC. For sleep stage distribution, the performance of YASA and Luna were negatively associated with % N1, STAGES and U‐Sleep were negatively associated with % N2, and STAGES was positively associated with % N3.

**FIGURE 5:**
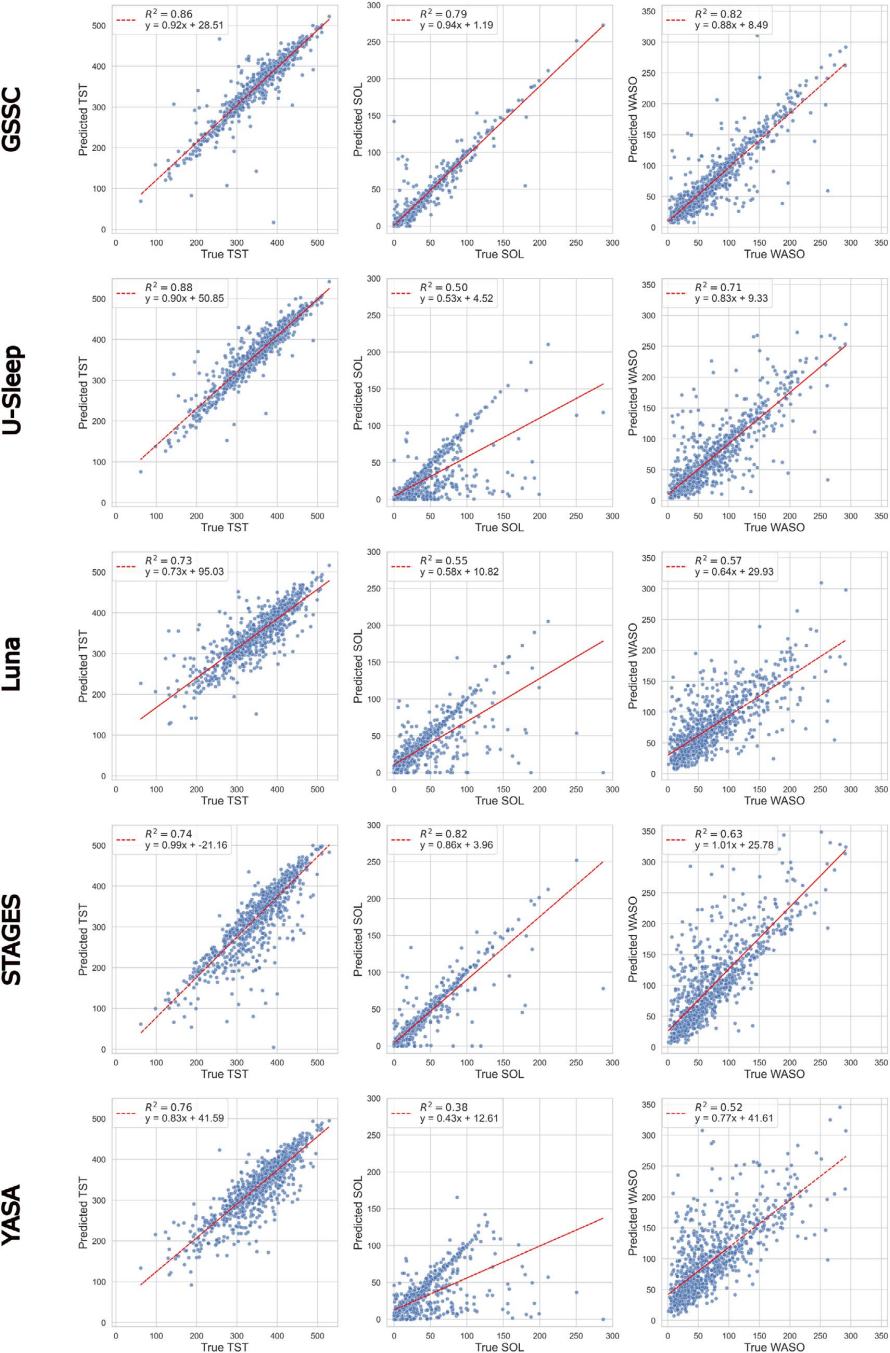
Scatter plots showing the correlation between true and estimated total sleep time (TST), sleep onset latency (SOL) and wake after sleep onset (WASO) based on sleep stage predictions from five different sleep stage classifiers (GSSC, U‐Sleep, Luna, STAGES, YASA).

Figure 5 shows scatter plots and linear regression fits when comparing sleep metrics (TST, SOL and WASO) estimated from the sleep stage predictions of each model with the gold‐standard sleep metrics calculated from technician‐based annotations. TST was best estimated with U‐Sleep and GSSC (*R* ^2^ = 0.88 and *R* ^2^ = 0.86, respectively), SOL was best estimated with STAGES and GSSC (0.82 and 0.79, respectively), and WASO was best estimated with GSSC (0.82). On the other hand, SOL and WASO were most poorly estimated with YASA (0.38 and 0.52), while all classifiers estimated TST well with *R* ^2^ > 0.7. Notably, U‐Sleep, Luna and YASA all showed a general tendency to overestimate SOL.

**FIGURE 6:**
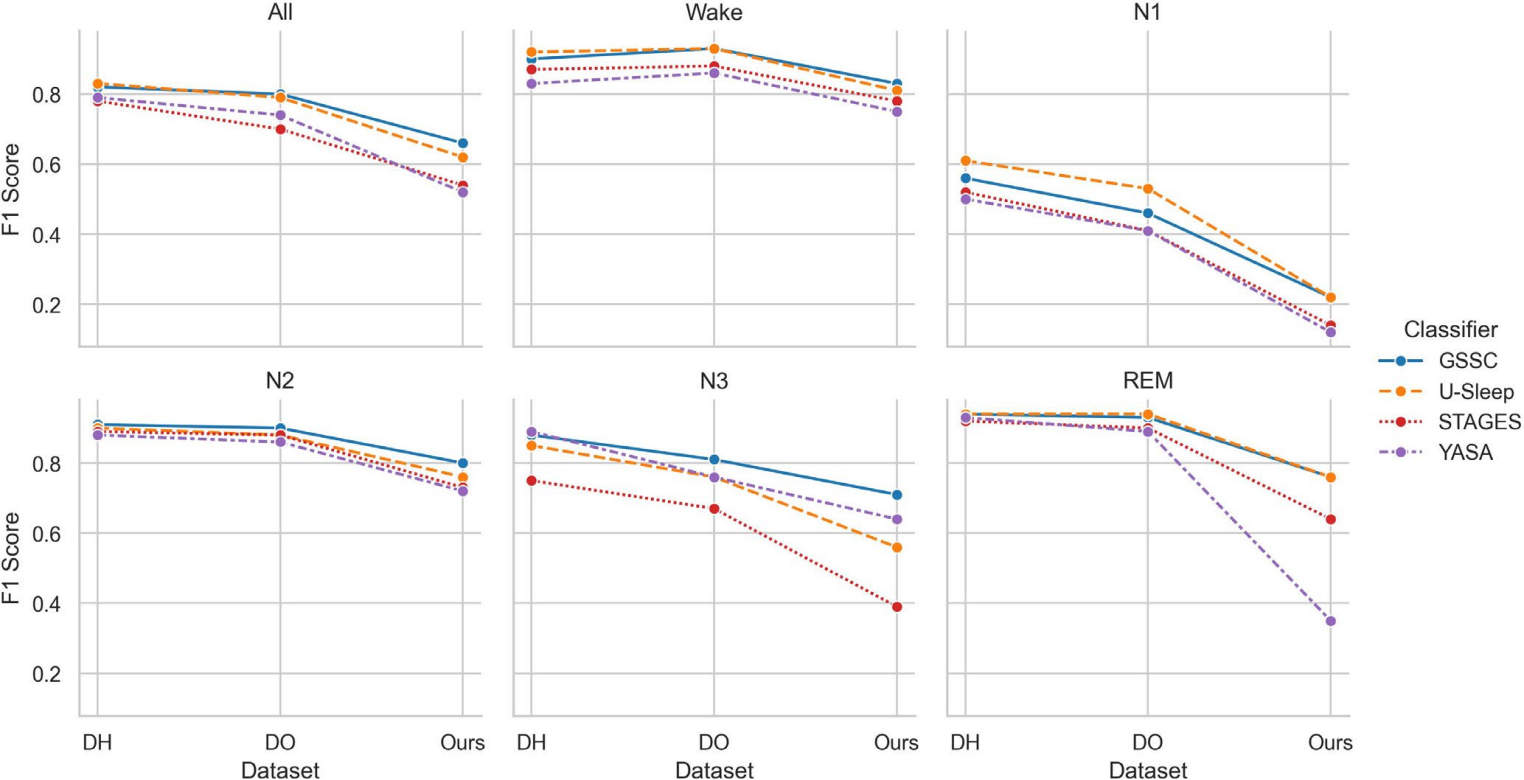
Performance comparison of four sleep staging classifiers (GSSC, U‐Sleep, STAGES and YASA) on three different datasets: DREEM healthy subjects (DH, *n* = 25), DREEM patients with sleep‐disordered breathing (DO, *n* = 55) and our dataset of patients with chronic insomnia (Ours, *n* = 904). Performance in the DREEM datasets is based on the consensus annotations of five expert scorers, whereas performance in our dataset is based on annotations from a single expert scorer.

Figure 6 shows the change in performance for each classifier in each sleep stage from the DREEM dataset (Guillot et al. 2020) (first healthy subjects, then patients with SDB) to our dataset of patients with chronic insomnia. Furthermore, Table S4 in the Supplement provides the performance values for each classifier and each dataset. There is a notable drop in the F1 score (~0.4) during N1 for all classifiers. Furthermore, in N3, there is a drop in the F1 score of 0.36 for STAGES, 0.29 for U‐Sleep and 0.25 for YASA, while in REM, there is a drop of 0.6 for YASA and 0.29 for STAGES. Comparatively, the drop in performance during Wake and N2 is small for all classifiers. For the macro F1, the smallest drop in performance is observed for GSSC, then U‐Sleep, while the largest drop is observed for YASA, then STAGES.

## 4 Discussion

This study provides the first comprehensive evaluation of five state‐of‐the‐art sleep stage classifiers—GSSC, U‐Sleep, Luna, STAGES and YASA—on a large, well‐labelled dataset of patients with chronic insomnia, with or without abnormal PSG results. While previous studies have assessed individual classifiers against polysomnography (https://zzz.bwh.harvard.edu/luna/ n.d.; Vallat and Walker 2021; Stephansen et al. 2018; Perslev et al. 2021; Hanna and Flöel 2023), this study uniquely applies the same dataset across all five classifiers, which were considered the leading performers in the field at the time of analysis. The findings reveal significant variability in performance across classifiers, particularly when assessing their ability to accurately classify individual sleep stages and estimate key sleep metrics. However, the results are based on a single dataset, scored by one technician and recorded using a single device. Further research on a more diverse set of data would allow testing the generalisability of our findings.

GSSC emerged as the most robust classifier, demonstrating the highest macro F1 score of 0.66 and superior performance across all individual sleep stages (Figure 2). Furthermore, GSSC showed the least amount of bias in terms of age, BMI and WASO (Figure 4), three key variables that are known to affect sleep architecture (Taillard et al. 2021; Yetton et al. 2018; Rao et al. 2009). This consistency underscores GSSC's potential as a reliable tool for automated sleep staging, particularly in clinical populations characterised by disturbed sleep patterns, such as those with chronic insomnia. While U‐Sleep followed closely with a macro F1 score of 0.62, its performance was more stage‐specific, excelling in detecting REM sleep but performing poorly in N3 (Figures 2 and 3), while providing the most accurate TST estimates yet severely underestimating SOL in many cases (Figure 5).

Interestingly, Luna and STAGES revealed contrasting strengths and weaknesses. Luna demonstrated strong sensitivity in identifying N3 (Figures 2 and 3), which may indicate its utility in studies prioritising deep sleep metrics, but it performed poorly in detecting light sleep stages, REM, and estimating SOL and WASO (Figure 5). On the contrary, STAGES showed reasonable performance across several stages and provided the most accurate SOL estimates but notably underperformed in N3 (Figure 2), with over half of N3 epochs misclassified as N2 (Figure 3). This misclassification highlights potential limitations when using STAGES in clinical contexts where accurate deep sleep detection is critical. Finally, YASA underperformed in most stages, particularly in N1 and REM (Figures 2 and 3), while also estimating SOL and WASO poorly (Figure 5), limiting its applicability for clinical or research settings requiring high precision. However, YASA demonstrated strong performance in detecting N2 and can provide sleep spindle detections, a hallmark of N2.

The confusion matrices (Figure 3) further highlight systematic patterns of misclassification shared by all classifiers. For example, N1 was frequently misclassified as Wake or N2, N3 was often misclassified as N2, while REM was commonly confused with Wake, N1, or N2 but never N3. These findings are consistent with technician‐based misclassifications reported in the literature (Lee et al. 2022; Arnal et al. 2020), and likely reflect inherent challenges in distinguishing subtle differences in EEG features between these stages, especially in populations with disrupted sleep architecture. However, these systematic misclassifications, particularly the frequent misclassification of N3 as N2, may also reflect bias introduced by using the scoring of a single technician, who might have favoured one scoring method over another for these specific stages. Importantly, no classifier showed confusion between REM and N3, indicating that all models successfully differentiate these biologically distinct stages.

The observed biases in classifier performance across demographic and sleep‐related variables (Figure 4) may arise from several factors. STAGES' better performance in females could reflect sex‐based differences in sleep architecture not fully captured in training data. Luna and STAGES' better performance in younger individuals may result from training datasets skewed toward younger participants, who typically exhibit more consistent sleep patterns. GSSC and U‐Sleep's lack of BMI bias suggests training on more diverse datasets. The absence of a relationship between classifier performance and normal versus abnormal PSG results in chronic insomnia patients may indicate insensitivity to this population's unique sleep disturbances. Subjective sleepiness positively influenced Luna, YASA and GSSC performance, likely due to clearer sleep–wake transitions in sleepier individuals, while STAGES and U‐Sleep's lack of association suggests less reliance on sleepiness‐related features. Positive associations with TIB, TST and % REM, and negative associations with ROL and WASO, reflect easier classification of longer, consolidated sleep with higher REM. YASA's weaker associations may indicate lower sensitivity to these metrics. These findings emphasise the need to consider demographic and sleep‐related factors when selecting and interpreting sleep staging classifiers, especially in diverse or clinically complex populations.

Lower performance was observed across sleep stages, particularly for N1, when testing the classifiers on our insomnia dataset compared to the DREEM (Guillot et al. 2020) dataset (Figure 6). This could be attributed to the unique characteristics of individuals with chronic insomnia, who experience fragmented sleep, increased arousals and heightened variability in sleep architecture, which can blur the boundaries between sleep stages and complicate automated sleep staging. Specifically, N1 represents a transitional stage between wakefulness and deeper sleep, and its classification often relies on subtle changes in EEG patterns, which may be less distinct in insomniac populations due to heightened cortical arousal and frequent wake–sleep transitions. However, the > 50% reduction in performance for N1 classification (Figure 6) is unlikely to be explained only by the subject characteristics; it may also be attributed to the use of a single technician, whose subjective scoring preferences could introduce bias, and/or the reliance on a single device for recording PSGs. Specifically, the use of the Nox A1 PSG system, which is likely not represented in the older cohorts of the NSRR used to train these algorithms, may have contributed to the reduced performance. Consequently, the lower overall performance compared to the DREEM dataset could reflect, among other factors, the reduced generalisability of the algorithms when applied to data recorded on a completely unfamiliar device.

In contrast, performance differences for other stages, such as Wake and N2, were less pronounced, as these stages are characterised by more robust and distinguishable EEG features, which may be less affected by the sleep fragmentation and hyperarousal observed in insomnia and may also be less prone to subjective choices in scoring by a technician. The large difference in performance during N3 and REM was more specific to classifiers such as STAGES and YASA (Figure 6), which were shown in other analyses to perform poorly in N3 and REM sleep, respectively (Figures 2 and 3). These findings underscore the challenges of applying automated sleep staging tools to clinical populations with atypical sleep architecture and highlight the need for classifiers trained or calibrated specifically on insomnia cohorts while including diverse recording equipment and technicians to improve performance.

While this study primarily focuses on the performance of different classifiers, several additional factors should be considered depending on the intended use case, such as ease of use, data sharing and privacy and functionality beyond sleep stage classification. For instance, U‐Sleep requires data to be uploaded to its server, which may pose challenges in applications with strict privacy or data security requirements. Similarly, STAGES and Luna demand significant programming expertise to configure the necessary environments and dependencies, limiting their accessibility. In contrast, YASA, despite its lower performance in this study, is highly accessible and user‐friendly, even for those with limited programming experience. Additionally, both YASA and Luna offer extended functionalities, including spindle detection, artefact identification and spectral analysis. While performance is a critical consideration, it is not the sole factor when selecting an appropriate classifier; practical considerations such as usability and additional features may also play a decisive role.

To our knowledge, this study is the first to evaluate the performance of these classifiers in the analysis of PSG for chronic insomnia. The findings affirm the potential value of PSG examinations in managing chronic insomnia. Historically, consensus recommendations have not included PSG as a diagnostic tool for insomnia, except in cases where other sleep pathologies, such as sleep apnea syndrome or restless legs syndrome, are suspected (*International Classification of Sleep Disorders* 2014). The definition of insomnia has traditionally focused exclusively on subjective complaints of poor sleep. For many years, clinical practice largely regarded overnight sleep recordings as adding little value to patient management. PSG criteria have not been widely used to propose or evaluate the efficacy of cognitive‐behavioural therapy for insomnia (CBT‐i), which remains a leading therapeutic option (Baglioni et al. 2014; Frase et al. 2023). Concerns have also been raised about the cost and feasibility of performing PSG for every patient presenting with chronic insomnia.

However, a meta‐analysis on the use of PSG in diagnosing insomnia, which analysed 23 studies with 582 patients with chronic insomnia (CI) and 485 good sleepers (GS), reported significant differences between these groups (Baglioni et al. 2014). Compared to GS, patients with CI slept approximately 23 min less, took an average of 6 min longer to fall asleep, and experienced about six more awakenings per night. These disruptions in sleep continuity resulted in a reduced sleep efficiency. Interestingly, patients with CI also showed a significant reduction in slow‐wave sleep and REM sleep compared to GS. Benz et al. recently reported a lack of agreement between subjective estimates of TST from PSQI sleep diaries and TST measured by PSG in both CI and GS (Benz et al. 2023). CI patients tended to underestimate their TST, while GS overestimated theirs. This finding suggests that relying solely on subjective assessments can misrepresent the severity of a patient's sleep disorder. In an initial analysis of a smaller subset of insomniac patients from our database, we highlighted that, beyond macroscopic sleep analysis, microstructural and spectral analyses of sleep stages provide deeper insights into different subjective experiences of sleep (Andrillon et al. 2020). For instance, EEG spectral analysis revealed that individuals with poor sleep misperception were more similar to CI patients than to GS. This underscores the importance of combining objective PSG metrics with subjective assessments to enhance the understanding and treatment of insomnia.

Our findings further challenge the additional value of PSG in the diagnosis and management of insomnia. However, they also highlight the necessity of identifying new biomarkers and defining insomnia subtypes for a more personalised approach to treatment. This need has been emphasised in recent studies (Blanken et al. 2019; Dikeos et al. 2023) and a position paper on insomnia and PSG (Frase et al. 2023). The emergence of advanced classifiers for analysing PSG data from insomnia patients provides a promising avenue for addressing these questions in the future.

Our study underscores at least four key reasons to analyse PSG data—whether acquired individually or collectively within groups—using classifiers: (1) efficiency: classifier‐based analysis is significantly faster and more cost‐effective compared to traditional visual data analysis; (2) reliability: certain classifiers, such as GSSC and U‐Sleep, demonstrate high reliability both in macroscopic sleep scoring and stage detection; (3) potential for improvement: the reliability of these classifiers is likely to improve further with advancements in artificial intelligence; and (4) comparative insights: utilising multiple classifiers allows us to compare clinical observations with diverse data‐driven approaches. This comparison enhances the robustness of insights, such as detecting stage N3 with Luna, REM sleep with U‐Sleep, or estimating sleep metrics with GSSC.

A limitation of this study is that recordings were only scored by a single technician, as opposed to other evaluation studies, where two or more technicians annotate the same recordings to allow for computation of interscorer variabilities and consensus annotations (Stephansen et al. 2018; Arnal et al. 2020; Danker‐Hopfe et al. 2009). Another notable limitation of this study is that the data were primarily recorded using the same equipment (NOX‐A1), resulting in limited variability in recording setups. Consequently, we were unable to assess the robustness of the evaluated classifiers across different hardware and software configurations. Variations in recording equipment, such as differences in amplifier sensitivity or electrode placement, can introduce variability in signal quality and characteristics, potentially impacting classifier performance. While this aspect could provide valuable insights into the generalisability of these tools, it was not the primary objective of this study. Instead, our focus was on evaluating classifier performance within a specific disease population of homogeneous patients with chronic insomnia. By maintaining consistency in recording equipment, we ensured that the observed differences in performance were attributable to the classifiers themselves rather than extraneous technical factors, aligning with our study's aim to better understand their utility in this specific clinical context.

As an extension of this study, future research should aim to develop and publish a dataset similar to the DREEM dataset but specifically tailored for patients with insomnia. A dataset like the one used in this study could serve as a foundation, though it would benefit from additional annotations provided by multiple technicians to enhance reliability and reduce potential bias. Future study should also focus on improving classifier accuracy through retraining on diverse datasets, incorporating multimodal data (such as respiratory signals and ECG) and addressing common misclassification patterns. Enhanced automated sleep staging tools have the potential to significantly improve the diagnosis and management of sleep disorders, paving the way for more personalised and effective treatment strategies.

## 5 Conclusion

This study provides a detailed comparison of five leading sleep stage classifiers in a challenging clinical population of patients with chronic insomnia. GSSC demonstrated superior overall performance, achieving the highest macro F1 score and sensitivity across most sleep stages while being minimally biased, making it the most reliable tool among those evaluated. U‐Sleep also performed well, particularly in estimating TST and detecting REM sleep, whereas Luna and STAGES showed mixed results, excelling in specific stages but underperforming in others. YASA underperformed in most stages, limiting its applicability in clinical and research settings. The observed misclassification patterns emphasise the ongoing challenges in automated sleep staging. These findings underscore the importance of selecting classifiers based on the specific needs of clinical or research applications. This study also highlights the potential interest of recording the sleep of patients with chronic insomnia for a more detailed analysis of biomarkers derived from PSG.

## Author Contributions

**Umaer Hanif:** conceptualization, investigation, writing – original draft, methodology, validation, visualization, writing – review and editing, software, formal analysis. **Anis Aloulou:** conceptualization, validation, writing – review and editing, data curation, resources. **Flynn Crosbie:** conceptualization, validation, writing – review and editing, data curation, resources. **Paul Bouchequet:** data curation, software. **Mounir Chennaoui:** data curation, software. **Thomas Andrillon:** writing – review and editing, validation, supervision. **Damien Leger:** conceptualization, funding acquisition, validation, writing – review and editing, project administration, data curation, supervision, resources.

## Conflicts of Interest

Dr. Hanif was an employee of BioSerenity.

Dr. Aloulou has no conflicts of interest to disclose.

Mr. Crosbie has no conflicts of interest to disclose.

Mr. Bouchequet has no conflicts of interest to disclose.

Dr. Chennaoui has no conflicts of interest to disclose.

Dr. Andrillon has no conflicts of interest to disclose.

Dr. Leger reports support to himself or his institution from Actelion, Bioserenity, Sanofi, Vanda, Linde, SOS Oxygene, and Vitalaire, including board membership, consulting or advisory roles, and travel reimbursement.

## Supporting information


**Table S1.** Characteristics of the included study sample. The characteristics are outlined for the two defined clinical subgroups: chronic insomnia (CI) with abnormal polysomnography (PSG) and CI with normal PSG. BMI—Body mass index, ESS—Epworth Sleepiness Scale, RLS—Restless legs syndrome, REM—Rapid eye movement, N1—Non‐REM stage 1, N2—Non‐REM stage 2, N3—Non‐REM stage 3.
**Table S2.** Mean F1 scores for each classifier and p‐values from pairwise comparisons in each sleep stage. Statistical differences were assessed using repeated measures ANOVA, followed by post hoc paired t‐tests for pairwise comparisons, with Bonferroni correction applied to adjust the p‐values. The number of comparisons was 60 (10 unique comparisons per sleep stage and 5 different sleep stages + all stages combined), and each p‐value was multiplied by this factor.
**Table S3.** Percentage/mean, beta and p‐value for each variable used to predict the macro F1 score of each sleep staging classifier using univariate linear regression models.
**Table S4.** Comparison of macro F1 scores for each classifier in each sleep stage on the DREEM dataset (first healthy subjects [DH], then patients with SDB [DO]) and on our dataset of patients with chronic insomnia (Ours).

## Data Availability

The data that support the findings of this study are available on request from the corresponding author. The data are not publicly available due to privacy or ethical restrictions.
