# Supplementary material for: Deciphering Insomnia: Benchmarking Automated Sleep Staging Algorithms for Complex Sleep Disorders
Source: J Sleep Res. 2025 Mar 27;34(6):e70048. doi: 10.1111/jsr.70048 (PMC12592834; doi:10.1111/jsr.70048)
Supplement: Supplementary file 1 — Table S1. Characteristics of the included study sample. The characteristics are outlined for the two defined clinical subgroups: chronic insomnia (CI) with abnormal polysomnography (PSG) and CI with normal PSG. BMI—Body mass index, ESS—Epworth Sleepiness Scale, RLS—Restless legs syndrome, REM—Rapid eye movement, N1—Non‐REM stage 1, N2—Non‐REM stage 2, N3—Non‐REM stage 3. Table S2. Mean F1 scores for each classifier and p‐values from pairwise comparisons in each sleep stage. Statistical differences were assessed using repeated measures ANOVA, followed by post hoc paired t‐tests for pairwise comparisons, with Bonferroni correction applied to adjust the p‐values. The number of comparisons was 60 (10 unique comparisons per sleep stage and 5 different sleep stages + all stages combined), and each p‐value was multiplied by this factor. Table S3. Percentage/mean, beta and p‐value for each variable used to predict the macro F1 score of each sleep staging classifier using univariate linear regression models. Table S4. Comparison of macro F1 scores for each classifier in each sleep stage on the DREEM dataset (first healthy subjects [DH], then patients with SDB [DO]) and on our dataset of patients with chronic insomnia (Ours). [file JSR-34-e70048-s001.docx]

Table S1. Characteristics of the included study sample. The characteristics are outlined for the two defined clinical subgroups: chronic insomnia (CI) with abnormal polysomnography (PSG) and CI with normal PSG. BMI – Body mass index, ESS – Epworth Sleepiness Scale, RLS – Restless legs syndrome, REM – Rapid eye movement, N1 – Non-REM stage 1, N2 – Non-REM stage 2, N3 – Non-REM stage 3.

| **Variable** | **CI w/ abnormal PSG** | **CI w/ normal PSG** |
| --- | --- | --- |
| Demographics | | |
| Sex (female) | 67.7% | 75.2% |
| Age (years) | 47.1 ± 14.4 | 40.9 ± 12.2 |
| BMI (kg/m^2) | 23.2 ± 3.9 | 22.8 ± 3.5 |
| Insomnia complaints and subjective sleepiness | | |
| Sleep initiation insomnia (0/1) | 54.4% | 59.7% |
| Sleep maintenance insomnia (0/1) | 77.6% | 74.2% |
| Early awakening insomnia (0/1) | 15.9% | 13.4% |
| ESS score (0-24) | 8.5 ± 5.4 | 7.6 ± 5.0 |
| Comorbidities | | |
| Depression (0/1) | 27.1% | 21.0% |
| Anxiety (0/1) | 19.6% | 20.8% |
| Cardiovascular disease (0/1) | 14.3% | 5.3% |
| Respiratory disease (0/1) | 5.2% | 4.8% |
| Gastrointestinal disease (0/1) | 4.2% | 4.3% |
| Infectious disease (0/1) | 1.2% | 0.5% |
| Metabolic disease (0/1) | 10.6% | 6.9% |
| Cancer (0/1) | 3.2% | 1.7% |
| Covid (0/1) | 3.0% | 2.9% |
| Epilepsy (0/1) | 0.7% | 1.1% |
| Pain (0/1) | 7.4% | 10.1% |
| Other comorbidity (0/1) | 38.3% | 37.8% |
| Substances and medication | | |
| Smoking (0/1) | 23.1% | 23.7% |
| Antidepressant (0/1) | 23.1% | 21.4% |
| Antihistamine (0/1) | 10.4% | 9.9% |
| Antipsychotic (0/1) | 3.8% | 3.8% |
| Benzodiazepine (0/1) | 25.9% | 25.3% |
| Melatonin (0/1) | 7.0% | 8.2% |
| Z-drugs (0/1) | 15.3% | 12.1% |
| Opioid (0/1) | 1.2% | 0.5% |
| RLS medication (0/1) | 1.7% | 0.5% |
| Other medication (0/1) | 41.6% | 41.8% |
| Sleep metrics | | |
| Time in bed (mins) | 459.7 ± 54.5 | 443.3 ± 50.8 |
| Total sleep time (mins) | 337.6 ± 70.3 | 397.1 ± 55.5 |
| Sleep onset latency (mins) | 38.5 ± 37.3 | 18.5 ± 24.2 |
| Wake after sleep onset (mins) | 83.7 ± 53.6 | 27.8 ± 23.7 |
| REM onset latency (mins) | 157.7 ± 80.6 | 116.9 ± 60.5 |
| N1 (%) | 4.4 ± 5.6 | 3.2 ± 2.9 |
| N2 (%) | 54.5 ± 12.1 | 51.4 ± 10.0 |
| N3 (%) | 23.2 ± 10.4 | 24.5 ± 9.1 |
| REM (%) | 17.9 ± 7.3 | 20.9 ± 7.0 |

Table S2. Mean F1 scores for each classifier and p-values from pairwise comparisons in each sleep stage. Statistical differences were assessed using repeated measures ANOVA, followed by post-hoc paired t-tests for pairwise comparisons, with Bonferroni correction applied to adjust the p-values. The number of comparisons was 60 (10 unique comparisons per sleep stage and 5 different sleep stages + all stages combined), and each p-value was multiplied by this factor.

| All stages | | | | | | |
| --- | --- | --- | --- | --- | --- | --- |
|  | **F1 score (**$\boldsymbol{\mu\pm\sigma}$**)** | **GSSC  (p-value)** | **U-Sleep  (p-value)** | **Luna  (p-value)** | **STAGES  (p-value)** | **YASA  (p-value)** |
| **GSSC** | 0.66 ± 0.11 |  | 2.82e-43 | 3.95e-169 | 6.39e-150 | 1.29e-264 |
| **U-Sleep** | 0.62 ± 0.12 |  |  | 2.79e-63 | 1.90e-97 | 4.00e-158 |
| **Luna** | 0.56 ± 0.11 |  |  |  | 2.50e-05 | 2.68e-47 |
| **STAGES** | 0.54 ± 0.13 |  |  |  |  | 2.81e-06 |
| **YASA** | 0.52 ± 0.11 |  |  |  |  |  |
| Wake | | | | | | |
|  | **F1 score** | **GSSC** | **U-Sleep** | **Luna** | **STAGES** | **YASA** |
| **GSSC** | 0.83 ± 0.16 |  | 4.17e-13 | 5.57e-69 | 8.37e-32 | 1.09e-101 |
| **U-Sleep** | 0.81 ± 0.16 |  |  | 1.68e-21 | 5.79e-07 | 1.24e-38 |
| **Luna** | 0.76 ± 0.17 |  |  |  | 5.92e-05 | 5.86e-04 |
| **STAGES** | 0.78 ± 0.17 |  |  |  |  | 5.98e-19 |
| **YASA** | 0.75 ± 0.17 |  |  |  |  |  |
| N1 | | | | | | |
|  | **F1 score** | **GSSC** | **U-Sleep** | **Luna** | **STAGES** | **YASA** |
| **GSSC** | 0.22 ± 0.16 |  | >0.999 | 2.16e-83 | 3.83e-60 | 3.65e-106 |
| **U-Sleep** | 0.22 ± 0.16 |  |  | 3.94e-90 | 2.12e-69 | 2.00e-105 |
| **Luna** | 0.14 ± 0.11 |  |  |  | 0.448 | 2.11e-03 |
| **STAGES** | 0.14 ± 0.13 |  |  |  |  | 2.86e-07 |
| **YASA** | 0.12 ± 0.10 |  |  |  |  |  |
| N2 | | | | | | |
|  | **F1 score** | **GSSC** | **U-Sleep** | **Luna** | **STAGES** | **YASA** |
| **GSSC** | 0.80 ± 0.11 |  | 3.42e-50 | 3.19e-108 | 7.11e-87 | 1.67e-150 |
| **U-Sleep** | 0.76 ± 0.12 |  |  | 1.10e-30 | 1.65e-19 | 3.95e-49 |
| **Luna** | 0.72 ± 0.13 |  |  |  | 0.185 | >0.999 |
| **STAGES** | 0.73 ± 0.12 |  |  |  |  | 1.33e-05 |
| **YASA** | 0.72 ± 0.12 |  |  |  |  |  |
| N3 | | | | | | |
|  | **F1 score** | **GSSC** | **U-Sleep** | **Luna** | **STAGES** | **YASA** |
| **GSSC** | 0.72 ± 0.20 |  | 5.82e-81 | >0.999 | 2.03e-166 | 1.22e-26 |
| **U-Sleep** | 0.56 ± 0.28 |  |  | 4.26e-69 | 1.75e-89 | 4.57e-27 |
| **Luna** | 0.71 ± 0.21 |  |  |  | 2.18e-160 | 7.98e-21 |
| **STAGES** | 0.39 ± 0.31 |  |  |  |  | 1.67e-137 |
| **YASA** | 0.65 ± 0.25 |  |  |  |  |  |
| REM | | | | | | |
|  | **F1 score** | **GSSC** | **U-Sleep** | **Luna** | **STAGES** | **YASA** |
| **GSSC** | 0.77 ± 0.19 |  | >0.999 | 2.08e-146 | 1.31e-44 | 1.90e-257 |
| **U-Sleep** | 0.77 ± 0.18 |  |  | 3.85e-141 | 7.00e-48 | 8.88e-264 |
| **Luna** | 0.48 ± 0.29 |  |  |  | 8.42e-37 | 3.71e-38 |
| **STAGES** | 0.64 ± 0.29 |  |  |  |  | 9.04e-103 |
| **YASA** | 0.35 ± 0.23 |  |  |  |  |  |

Table S3. Percentage/mean, beta, and p-value for each variable used to predict the macro F1 score of each sleep staging classifier using univariate linear regression models.

| Variable | Value | GSSC  F1 score | U-Sleep  F1 score | Luna  F1 score | STAGES  F1 score | YASA  F1 score |
| --- | --- | --- | --- | --- | --- | --- |
| Sex (male) 30.4% | Beta | -0.011 (-0.028, 0.005) | -0.009 (-0.026, 0.008) | -0.001 (-0.017, 0.015) | -0.024 (-0.043,  -0.005) | -0.015 (-0.030, 0.001) |
|  | p-value | 0.168 | 0.299 | 0.907 | 0.0129 | 0.0646 |
| Age (years) 45.7 ± 14.1 | Beta  (10 years) | -0.011 (-0.013,  -0.010) | -0.014 (-0.016,  -0.012) | -0.024 (-0.025,  -0.022) | -0.023 (-0.025,  -0.021) | -0.018 (-0.019,  -0.016) |
|  | p-value | 2.51e-05 | 6.64e-07 | 2.05e-20 | 2.43e-13 | 3.48e-12 |
| BMI (kg/m^2^) 23.1 ± 3.84 | Beta  (5 units) | -0.002 (-0.006, 0.003) | -0.004 (-0.008, 0.001) | -0.017 (-0.021,  -0.013) | -0.015 (-0.020,  -0.010) | -0.009 (-0.013,  -0.005) |
|  | p-value | 0.725 | 0.499 | 5.84e-04 | 0.0119 | 0.0530 |
| Abnormal PSG (0/1) 76.3% | Beta | 0.010 (-0.008, 0.027) | -0.005 (-0.023, 0.013) | 0.006 (-0.011, 0.024) | -0.010 (-0.031, 0.011) | 0.003 (-0.014, 0.020) |
|  | p-value | 0.269 | 0.610 | 0.463 | 0.341 | 0.741 |
| ESS (0-24) 8.28 ± 5.34 | Beta  (3 units) | 0.005 (0.002, 0.009) | 0.002 (-0.002, 0.005) | 0.007 (0.003, 0.010) | 0.002 (-0.002, 0.005) | 0.006 (0.002, 0.009) |
|  | p-value | 0.0926 | 0.571 | 0.0219 | 0.665 | 0.0549 |
| TIB (mins) 456 ± 54.0 | Beta  (30 units) | 0.007 (0.006, 0.008) | 0.005 (0.004, 0.005) | 0.006 (0.005, 0.007) | 0.006 (0.005, 0.007) | 0.002 (0.002, 0.003) |
|  | p-value | 8.63e-04 | 0.0348 | 0.00436 | 0.0152 | 0.221 |
| TST (mins) 352 ± 71.7 | Beta  (30 units) | 0.006 (0.006, 0.007) | 0.010 (0.009, 0.010) | 0.007 (0.007, 0.008) | 0.009 (0.008, 0.009) | 0.005 (0.004, 0.006) |
|  | p-value | 7.21e-05 | 2.19e-09 | 1.93e-06 | 3.31e-06 | 0.00102 |
| log(SOL (mins) + 1) 3.05 ± 1.09 | Beta  (1 unit) | 0.008 (0.002, 0.015) | -0.002 (-0.009, 0.005) | 0.010 (0.003, 0.017) | 0.002 (-0.006, 0.010) | 0.006 (-0.001, 0.013) |
|  | p-value | 0.0150 | 0.593 | 0.00435 | 0.646 | 0.0753 |
| log(WASO (mins) + 1) 3.98 ± 0.811 | Beta  (1 unit) | -0.009 (-0.018,  -0.000) | -0.020 (-0.029,  -0.010) | -0.011 (-0.020,  -0.002) | -0.016 (-0.027,  -0.006) | -0.012 (-0.021,  -0.003) |
|  | p-value | 0.0492 | 4.47e-05 | 0.0217 | 0.00276 | 0.00858 |
| ROL (mins) 148 ± 78.2 | Beta  (10 units) | -0.002 (-0.002,  -0.002) | -0.003 (-0.003,  -0.002) | -0.001 (-0.001,  -0.001) | -0.002 (-0.003,  -0.002) | -0.001 (-0.001,  -0.001) |
|  | p-value | 5.66e-05 | 3.89e-08 | 0.0515 | 3.19e-05 | 0.0322 |
| log(N1 (%) + 1) 1.35 ± 0.733 | Beta  (1 unit) | -0.009 (-0.019, 0.001) | -0.005 (-0.016, 0.005) | -0.011 (-0.021,  -0.001) | -0.009 (-0.021, 0.002) | -0.012 (-0.022,  -0.002) |
|  | p-value | 0.0761 | 0.341 | 0.0275 | 0.120 | 0.0149 |
| N2 (%) 53.7 ± 11.7 | Beta  (5 units) | 0.000 (-0.001, 0.002) | -0.002 (-0.004,  -0.001) | -0.001 (-0.003, 0.000) | -0.006 (-0.008,  -0.004) | 0.001 (-0.000, 0.002) |
|  | p-value | 0.795 | 0.202 | 0.451 | 0.00119 | 0.500 |
| N3 (%) 23.5 ± 10.1 | Beta  (5 units) | -0.001 (-0.002, 0.001) | -0.001 (-0.003, 0.001) | 0.001 (-0.000, 0.003) | 0.007 (0.005, 0.009) | 0.001 (-0.000, 0.003) |
|  | p-value | 0.750 | 0.628 | 0.442 | 0.00219 | 0.546 |
| REM (%) 18.6 ± 7.30 | Beta  (5 units) | 0.010 (0.008, 0.013) | 0.015 (0.013, 0.017) | 0.010 (0.007, 0.012) | 0.011 (0.009, 0.014) | 0.004 (0.002, 0.006) |
|  | p-value | 6.44e-05 | 1.05e-08 | 1.73e-04 | 2.08e-04 | 0.101 |

Table S4. Comparison of macro F1 scores for each classifier in each sleep stage on the DREEM dataset (first healthy subjects [DH], then patients with SDB [DO]) and on our dataset of patients with chronic insomnia (Ours).

| Stage | GSSC F1 (DH) | GSSC F1 (DO) | GSSC F1 (Ours) |
| --- | --- | --- | --- |
| All | 0.82 | 0.80 | 0.66 |
| Wake | 0.90 | 0.93 | 0.83 |
| N1 | 0.56 | 0.46 | 0.22 |
| N2 | 0.91 | 0.90 | 0.80 |
| N3 | 0.88 | 0.81 | 0.71 |
| REM | 0.94 | 0.93 | 0.76 |
|  | U-Sleep F1 (DH) | U-Sleep F1 (DO) | U-Sleep F1 (Ours) |
| All | 0.83 | 0.79 | 0.62 |
| Wake | 0.92 | 0.93 | 0.81 |
| N1 | 0.61 | 0.53 | 0.22 |
| N2 | 0.90 | 0.88 | 0.76 |
| N3 | 0.85 | 0.76 | 0.56 |
| REM | 0.94 | 0.94 | 0.76 |
|  | STAGES F1 (DH) | STAGES F1 (DO) | STAGES F1 (Ours) |
| All | 0.78 | 0.70 | 0.54 |
| Wake | 0.87 | 0.88 | 0.78 |
| N1 | 0.52 | 0.41 | 0.14 |
| N2 | 0.89 | 0.88 | 0.73 |
| N3 | 0.75 | 0.67 | 0.39 |
| REM | 0.92 | 0.90 | 0.64 |
|  | YASA F1 (DH) | YASA F1 (DO) | YASA F1 (Ours) |
| All | 0.79 | 0.74 | 0.52 |
| Wake | 0.83 | 0.86 | 0.75 |
| N1 | 0.50 | 0.41 | 0.12 |
| N2 | 0.88 | 0.86 | 0.72 |
| N3 | 0.89 | 0.76 | 0.64 |
| REM | 0.93 | 0.89 | 0.35 |
